# Observations on the Sanitary Condition with Remarks on the Pathology and Treatment of Some of the Diseases of Southern Indiana

**Published:** 1871-04

**Authors:** Robert Robson

**Affiliations:** New Harmony, Indiana


### OBSERVATIONS ON THE SANITARY CONDITION, WITH REMARKS ON THE PATHOLOGY AND TREATMENT OF SOME OF THE DISEASES, OF SOUTHERN INDIANA.

By ROBERT ROBSON, M.D., New Harmony, Indiana.

The following remarks on the sanitary condition of the Valley of the Wabash, and on some of the diseases of Southern Indiana, are submitted, under the conviction, that it is the duty of every physician to do something to elevate the character and increase the usefulness of his profession, and, although they may not contain ought that is new, nevertheless, as history of the past, they may, by comparison, throw some light on the coming events of the future.

If we ever have a digest worthy to serve as a guide to the practice of medicine in Indiana, its best materials will undoubtedly be sought in the accumulated contributions of local medical history; and certainly, no State, from its great extent and varied natural features, calls more imperiously for a careful observation of facts than our own.

It is a frequent remark of the present time, that however rapidly men advance in an intellectual point of view, they still fall short, physically and morally, of what they ought to be; that mankind is obnoxious to many more diseases now than formerly. That there is much, on a hasty survey, which seems to countenance such complaints, especially in the excessive refinement of manners, and the luxuries attendant on civilization, from whence effeminacy and debility supervene, the increase of diseases, and the endless variety of the means of production are so apparent.

This is certainly not the case, but wholly without foundation.

During the first quarter of a century of my medical life in my present locality, we undoubtedly had more diseases in less than half of our present population. At that early period, the County was uncultivated, the inhabitants poor, living in miserable cabins, surrounded by timber and marshes, and exposed to a vitiated atmosphere; but, with the increase of our population during the last twenty years (for I have occupied my present position forty-five years) the sanitary condition of Southern Indiana, and the improvement of its inhabitants, are truly wonderful; not only have diseases decreased in number and intensity, but every onward step in the path of intellectual progress has had a beneficial influence on our population. It cannot be denied, that with the progress of civilization, not only does the population in general increase in health and prosperity, but longevity also, whilst the liability to sickness, and to the sufferings to which all are liable, are materially lessened.

Great improvements have been made in the comforts and living of our people, and, in the dress of the community also; though some things still remain to be done in reference to the female portion of it. All those articles that formerly interfered with the free play of the organs are falling into discredit. Cleanliness is now much more attended to than formerly—the important influence of the skin on the health of the entire system is now felt and appreciated as it deserves. The necessity of free ventilation and wholesome air is duly recognized, and the facilities of procuring wholesome food, are now much increased through the improved state of agriculture, all of which, besides very many other improvements not mentioned, tend to the comfort, and promise permanent advantage to our people. They prevent the spread as well as the production of disease.

The progress of civilization, and the improvements of practical medicine, have vastly contributed to the decrease of our diseases—reference to historical and statistical accounts of almost all diseases, will satisfy any one of the truth of this position.

Among the diseases to which we are subject during the winter, stands first and foremost, pneumonia and its complications. It prevails from October to April or May, some seasons it is very prevalent, amounting almost to an epidemic. The symptoms of pneumonia simplex present but little variation. The disease is ushered in by a chill, which is soon followed by fever, flushed face, headache, and the signs of local determination of blood. Unless the case is complicated with pleurisy, the patient complains of a dull, aching pain in the chest, quick pulse, and high fever, the probable result of inflammatory congestion of some portion of the lungs. In some cases dyspnœa is urgent, compelling the patient to lie on his back with his shoulders elevated. This I conceive to be the result of the congestion and consecutive exudation which diminishes the field of hematosis, and lessens the amount of oxygen entering the blood. The increase of fibrin diminishes the red globules of the blood, which are the bearers of oxygen to the tissues, and consequently lessens the quantity of blood in the circulation, and gives rise to dyspnœa. Besides, the more intense the fever, the more active the combustion, the greater the consumption of oxygen; therefore, the intensity of the dyspnœa is in proportion to the activity of the fever. The pulse, in such cases, is seldom less than 100, and more frequently 120 or more. The fever, in this locality, is generally of a high grade of action. During the first stage—that of congestion—there is but little expectoration, but, as the disease assumes the second stage—that of exudation—the expectoration becomes more abundant, and is most generally of a rusty, semi-transparent, tenacious, and coherent character. The tongue is often furred, the urine high-colored, the bowels costive, and the patient sometimes, though seldom, delirious.

In simple pneumonia, the physical signs are very generally well-marked; dulness on percussion, and crepitation on auscultation.

In pleuro-pneumonia the general symptoms differ but little from the above, with the exception of an acute pain in the side, making respiration, coughing, etc., exceedingly painful.

The treatment, in such cases, has for many years consisted of calomel, Dover's powder, antimonials, digitalis, blisters, etc.; a mixed treatment, anti-febrile, modifying the temperature and the pulse, etc., resulting, probably, in a mortality of 15 or 20 per cent., in my humble opinion. I would observe, that should the lungs, after the above treatment, still remain unresolved, and the strength of the patient failing, our next recourse would then be to stimulants and tonics, to aid in bringing about a resolution of the existing exudation, but I do not propose to enter into the detail of treatment, as it is supposed that every physician who can treat a severe case successfully, will have sufficient discretion and judgment to adjust his practice to the milder forms.

We learn from the writings of Prof. Jaccoud, at the Hospital de la Charrite, in Paris, Published in the *Richmond and Louisville Medical Journal,* that such remedial agents are only applicable to symptoms, and are only admissable during the first 48 hours, that they do not cut short or abridge the disease in any way, but depress the nervous system, produce nausea, lessen the pulse, and increase the renal and cutaneous secretions, and by increasing the depression produce collapse, or create a quasi paralytic condition of the system, which plunges the patient into an artificial adynamia.

Such treatment, he says, will neither hasten the resorption of the hepatization, or diminish the duration of the disease.

It has repeatedly been said that tart. antimon. possessed the property of favoring liquification and resorption of the pulmonary exudation, and thus abridge the duration of the disease.

This, he says, is not so. We are informed in reference to the natural march of pneumonia, that hundreds of cases let alone, demonstrate that resolution from the fifth to the seventh day is the rule. Continue the tart. antimon. over the time specified above, and you have the toxic action. That pneumonia treated by bleeding has the largest mortality, by the tart. antimon. the next largest, and by the mixed treatment still less, though large, *and by tonic medication the least.*

The published statistics show conclusively that the least mortality is obtained with the exclusively tonic treatment, that is to say, with sulph. quin., wine, and a mild diet, even during the height of the disease. This is one of the most remarkable of results. The small mortality of three per cent. proves the treatment innocuous. Although tart. antimon, digitalis, etc., are not such serious inconveniences as bleeding, yet the state of many patients do not bear even this temporary draught upon the forces of the organism. If I am not mistaken, Robert Bently Todd introduced the treatment of pneumonia by alcohol, even during the achme of the fever, while Prof. Jaccoud used it only in adynamic cases. I have treated several cases of pneumonia in strict accordance with the views laid down in the work of Prof. Jaccoud, and certainly so far I have no reason to regret it, but, on the contrary, believe them to indicate the true treatment in all such cases. Alcohol, in any of its forms, exercises an energetic stimulation upon the nervous system, and as it is precisely the collapsed state of the nervous system that we have to contend against, it is invaluable as a stimulant in adynamic excitation of the nerves, and also as a modifier of the nutritive combustion which is injurious in such cases. The *modus operandi* is, that the excessive congestion pertinent to fever is partly entertained at the expense of the alcohol absorbed, instead of being kept up at the expense of the organic substance itself. In this way alcohol becomes a saving agent, by restricting the destruction of other combustible material in the organism. The part not eliminated remains in the system, and is consumed by oxygen gas.

Bleeding, so usual a few years since in this section, is now seldom resorted to in any of the chest diseases. We are not often called upon in the early stage of disease, when the use of the lancet would be a powerful auxiliary in modifying the inflammatory congestion consequent on the first stage of pneumonia, etc., etc.

Neuralgia has been of frequent occurrence during the winter, and commanded considerable attention at the hands of the practicing physician.

Though the doctrine of spinal neuralgia is founded on anatomy and physiology, and observation and experience daily adding their sanction to the deduction of science, yet, notwithstanding, there is no subject that is involved in so much obscurity as that of the true pathology of neuralgia, and certainly none that requires at our hands a more thorough investigation, with the view to determine, if possible, its seat, its essential nature, and best methods of cure. Whether it is an idiopathic affection of the nervous centres and branches, or is merely sympathetic of remote organic irritation, or both, it must be admitted that some of the most prominent phenomena of neuralgia, in many cases at least, indicate the nervous centres, especially the spinal marrow and its branches, to be the original seat of the disease, yet painful diseases of the viscera are, it is well known, very often accompanied with pain in a corresponding point of the vertebral column. Cramps of the stomach, when at their height, give rise to pain, more or less acute, about the fourth cervical vertebra, and the patient often complains more of the pain than of the cramp of the stomach. Severe functional diseases, in any of the viscera, may so influence the nerves of that organ,—for example, take the headache from disordered stomach, the par vagum which arises from the brain, and is distributed to the stomach, is irritated, and pain is immediately felt at its origin. The form of neuralgia which most appears in the Wabash bottoms, is generally facial in character, and of undoubted miasmatic origin. I have even regarded this form of neuralgia as an intermittent, and treated it invariably with success by a mercurial cathartic, quinine and iron, as the best remedial agents to which this affection most readily yields. The female portion of the population living in the valley of the Wabash river are evidently more subject to this affection than males. This may be owing to the peculiar delicacy of the nervous system by which they are more susceptible to impressions, or perhaps to the suffering consequent on parturition, etc. In the early part of my professional life, emboldened by high authority, I treated every case of neuralgia by bleeding, or cuping, or counter-irritation over the spinal column by means of blisters, tart. antim. ointment, etc., etc., which, while it released many, failed to relieve others, to whom the tonic treatment of quinine and iron are administered with success.

I have lately witnessed two of the most violent cases of neuralgia, in the town of New Harmony, which yielded, after much previous suffering, to the hydrate of chloral, which I will report as soon as time will permit.

